# Factors affecting outdoor time and screentime in the context of preschool myopia prevention: a mixed-methods study

**DOI:** 10.1038/s41433-026-04625-8

**Published:** 2026-06-10

**Authors:** Simran Khutan, Rebecca J. McLean, Mervyn G. Thomas, Sohaib R. Rufai

**Affiliations:** The University of Leicester Ulverscroft Eye Unit, Leicester Royal Infirmary, Leicester, UK

**Keywords:** Outcomes research, Epidemiology, Refractive errors, Risk factors

The “myopia epidemic” represents a significant public health concern, with the global prevalence estimated to reach 52% by 2050 [1]. Earlier onset of myopia is associated with an increased risk of developing high myopia and related complications in adulthood [2].

Studies have shown that spending 13-h outdoors per week is protective against myopia [3], whilst increased screentime is a risk factor. However, little is known about these behaviours in preschool children. This study aims to explore these phenomena in the context of preschool myopia prevention.

A cross-sectional, mixed-methods study was conducted in Leicestershire, UK, representing the first phase of the COSMIC Study (Correlating Outdoor time, Screentime and Myopia in preschool Children). A semi-structured focus group discussion was held in January 2025, followed by the distribution of an online survey to parents of children aged 1–4 years between March and April 2025. Survey distribution was via seven consenting preschool centres (47% recruitment rate) and social media. Closed and open-ended questions were combined with Likert scales to explore outdoor time and screentime behaviours. Statistical analysis was performed on quantitative data using Stata 18.0 (StataCorp LLC, Texas, USA). Qualitative data was analysed through thematic analysis and aimed to explore the supportive and hindering factors to pre-school children meeting outdoor and screentime guidelines.

Of 153 parents invited to complete the survey, fifty-two responses were received; a response rate of 34% (mean child age: 2.7 years; SD ± 0.97; range: 1.2-4.9 years). Only five children (9.6%) were reported to spend two hours per day outdoors, thus meeting outdoor recommendations [3]. Despite this low outdoor adherence, Likert scale responses revealed all parents believed their child’s outdoor time to be of importance, and a greater proportion reported these guidelines to be realistic (Fig. 1). Parents were generally unaware of the protective effect of outdoor time for myopia prevention.

**Fig. 1:**
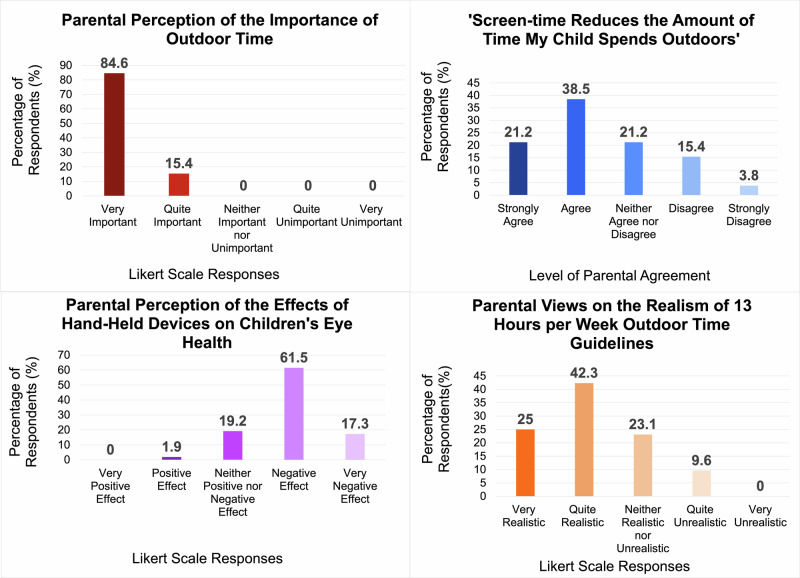
Likert scale responses to the following questions. **a** How important do you believe it is for your child to spend time outdoors regularly at their current age?. **b** To what extent do you agree with the following statement, ‘Screen-time reduces the amount of time my child spends outdoors?’. **c** In general, do you think that children spending time on hand-held screens has a beneficial or negative effect on their eye health?. **d** How realistic do you feel spending 13 h per week outside is for your child?.

Conversely, 36 children (78.3%) were reported to meet screentime guidelines [4]. Only one child was reported to be myopic. Statistical analysis between outdoor time and screentime showed no significant association (Fisher’s Exact test, *p* = 0.30).

Thematic analysis revealed five themes, displayed in Table 1.

**Table 1:** Five Key Themes Derived from Thematic Analysis.

Themes	Sample responses	Theme description
1. Environmental and structural influences	“If you do send your child to clubs on Saturdays, its indoors, so it’s limited the activities that they can do. What can you do with them other than go for a walk?”	Parents feel that the availability and accessibility of nearby outdoor spaces play a predominant role on their child’s outdoor time. They express difficulty in locating forms of outdoor play that cater to pre-school children.
2. Parenting beliefs, emotions and perceived influence	“… like these kids are very demanding and they do want your one-on-one time. For your own mental health, I feel like you can only give so much…”	Many parents feel the strain of household chores, responsibilities and work commitments, alongside emotional guilt and overwhelm when their child is unable to meet recommended guidelines.
3. Screen-time as a practical tool	“… so we have also used apps and videos to expose [child] to Gujarati, he isn’t bilingual, but he wouldn’t know any if it wasn’t for that content.”	Parents recognise the importance of screen use on their child’s education, though there is an increased reliance on screens as a distraction to alleviate parenting demands.
4. Societal and cultural norms	“Our lives are so integrated with screens and laptops now. Is that just something that to some extent needs to be accepted?”	Parents express a loss of control surrounding the direction of daily routines towards the use of digital devices, with feelings of tension between keeping their child aligned with modernity and balancing the harmful effects of digital devices.
5. Health effects influencing parental behaviours	“[child’s] attention span is shorter indoors…he’s completely reliant on the stimulation of things, whereas outdoor the environment is stimulating enough.”	Outdoor time is perceived by parents to have positive outcomes on their child’s overall health. Conversely, parents associate increased screentime with bad behaviour, often leading to screentime restrictions.

This study offers fundamental insights into screentime and outdoor behaviours within a previously understudied cohort. Although few children met outdoor recommendations (*n* = 5, 9.6%) compared to screentime guidelines (*n* = 36, 78.3%), most parents (*n* = 35, 67.3%) perceived weekly outdoor recommendations as ‘very realistic’ or ‘realistic’ (*n* = 13, 25%; *n* = 22, 42.3% respectively). Limited parental awareness of the benefits of outdoor time for myopia prevention – revealed through thematic analysis – possibly explains the disparity between screentime and outdoor adherence and thus offers a potential target in myopia prevention strategies. We also suspect that the amount of screentime may have been underestimated or underreported.

To the best of our knowledge, this study represents the first British study to provide insights into preschool children’s outdoor and screentime behaviours in relation to myopia and relevant guidelines. Findings demonstrate an importance in reducing screentime and increasing outdoor time within the pre-school population and offer a foundational understanding for future research in targeting such behaviours throughout myopia prevention.

## Data Availability

Data available upon reasonable request
